# Maternal deaths associated with hypertension in South Africa: lessons to learn from the Saving Mothers report, 2005–2007

**DOI:** 10.5830/CVJA-2010-042

**Published:** 2011-02

**Authors:** J Moodley

**Affiliations:** Women’s Health and HIV Research Group, Nelson R Mandela School of Medicine, University of KwaZulu-Natal, Durban, South Africa

**Keywords:** audit, maternal mortality, hypertension, lessons to learn

## Abstract

**Summary:**

From 2005–2007, there were 622 deaths associated with hypertensive disorders of pregnancy. Eclampsia was the major cause of death (*n* = 344; 55.3%). There were 173 (28.3%) deaths due to pre-eclampsia, and 38 (6.1%) associated with chronic hypertension. Cerebral complications were the final cause of death in 283 (45.5%), while cardiac failure and respiratory failure were the final causes in 142 (22.8%) and 158 (25.4%), respectively.

Major problems were identified in all areas of assessment. Non-attendance for antenatal care (*n* = 106; 19.4%) and delay in seeking help (*n* = 106; 19.4%) were major patient-related factors. Communication problems (*n* = 63; 10.8%) and lack of facilities (*n* = 50; 8.5%) were health administration issues. Health worker-avoidable factors included problem recognition, delay in referral and management at an inappropriate level of healthcare.

Compared to the previous report of 2002–2004, there was a reduction in deaths due to hypertension.

## Summary

Hypertensive disorders of pregnancy (HDP) are not only the commonest medical complication in pregnancy, but remain the commonest direct cause of maternal mortality in South Africa. In the United Kingdom, despite the fact that maternal deaths are uncommon, hypertensive deaths are the second commonest cause after thrombo-embolism.^1^ The latest Saving Mothers report (2005–2007) indicates that there were 622 maternal deaths from hypertensive disorders, virtually the same as in the previous report of 2002–2004.^2^.^3^ The number of deaths reported in the two previous Saving Mothers reports were 1999–2001: *n* = 507^4^ and 2002–2004: *n* = 628.^3^ The difference in numbers between the first two reports probably indicated under-reporting in the triennium 1999–2001. The two most recent reports, 2002–2004^3^ and 2005–2007,^4^ provide a better reflection of the numbers of maternal deaths from HDP.

All maternal deaths in South Africa are notifiable to the Department of Health and are reported in a structured data form called the Maternal Death Notification Form, which contains demographic data and clinical details surrounding the primary and secondary causes of death. This form, together with a copy of the hospital records, is sent to the provincial Maternal, Women and Child Cluster who in turn, get an experienced maternal health specialist and a midwife (trained assessors) to formulate a confidential opinion on any substandard care based on patient-related, administrative, and health personnel-related factors. All assessments are collated every three years and published as the Saving Mothers report. Recommendations to reduce maternal deaths are also included in this report.

## Causes of maternal deaths in South Africa

The primary causes of deaths in the various sub-categories of hypertensive disorders are shown in Table 1. The primary causes are similar to the 2002–2004 report.^3^ The numbers of deaths assigned to the HELLP syndrome declined in the latest report; 54 compared to 70 in the 2002–2004 report.

**Table 1.** Primary Obstetric Causes Of Death In The Sub-Categories

	*2005–2007*		*2002–2004*	
Sub-categories	n	%	n	%
chronic hypertension	38	6.1	37	5.9
proteinuric hypertension	173	27.8	171	27.2
eclampsia	344	55.3	347	55.3
HELLP syndrome	54	8.7	70	11.1
rupture of the liver	10	1.6	3	0.5
acute fatty liver	3	0.48	0	0.0
Total	622		628	

There was a small decline in the number of deaths from cerebral complications, from 316 (2002–2004) to 283 patients. Nonetheless, deaths from cerebrovascular events are a serious concern. Guidelines for the treatment of hypertension associated with severe pre-eclampsia and eclampsia are provided by the National Health Department. Deaths from intracranial haemorrhage probably indicate inadequate treatment of severe hypertension or patient delay in seeking help. In the latest Why Women Die (confidential enquiry into maternal deaths in the UK, 2003–2005),^1^ the single major failing in clinical care in pre-eclampsia was inadequate treatment of sustained systolic hypertension.

There was an increase in the number of deaths from cardiac failure as shown in Table 2. On the other hand, deaths from renal failure have continued to decline through the three Saving Mothers reports: 1999–2001 (*n* = 90; 17.8%), 2002–2004 (*n* = 88; 14.8%), and 2005–2007 (*n* = 64; 10.3%).^2^,^3^ It is, however, difficult to distinguish what is being reported when patients are assessed to have died from cardiac, renal or respiratory failure. Cardiac failure may lead to hypotension or pulmonary oedema or both; renal failure implies fluid overload and therefore pulmonary oedema. Respiratory failure also carries the inference of tachypnoea that may develop for many reasons, including pulmonary oedema. It is likely that all these terms may indicate deaths due to fluid overload, manifesting as pulmonary oedema. If this is the case, then the total number of deaths due to this cause exceeds the number of women dying from cerebral events, and the number of such deaths has increased during the most recent triennium, whereas the neurological mortality has decreased.

**Table 2.** Final And Contributory Causes Of Maternal Deaths For Hypertension And A Comparison With 2005–2007 And 2002–2004

*Organ system*	*2005–2007*		*2002–2004*	
	n	*%*	n	*%*
Hypovolaemic shock	50	8.0	49	7.8
Septic shock	20	3.2	16	2.5
Respiratory failure	158	25.4	155	24.7
Cardiac failure	142	22.8	89	14.2
Renal failure	64	10.3	88	14.8
Liver failure	30	4.8	31	4.9
Cerebral complications	283	45.5	316	50.3
Metabolic complications	26	4.2	7	1.1
DIC	70	11.3	89	14.2
Multi-organ failure	88	14.1	104	16.6
Immune system failure	31	5.0	18	2.9
DIC: Unknown	56	9.0	56	8.9

## Demographic data

Table 3 lists the age distribution and deaths due to hypertension. Of the 344 deaths from eclampsia, the majority occurred in the age group below 34 years. However, there remains a large proportion of deaths in the age group ≥ 35 years. Most women who die due to eclampsia and proteinuric hypertension are of low parity, namely, 0 and 1.

**Table 3.** Age Distribution And Death Due To Hypertension In Pregnancy (Years)

*Category*	*< 20*	*20–24*	*25–29*	*30–34*	*35–39*	*40–44*	*45+*	*Unknown*	*Total*
Chronic hypertension	0	3	6	7	12	8	2	0	38
Proteinuric hypertension	19	43	33	44	21	11	2	0	173
Eclampsia	83	88	62	60	39	10	1	1	344
HELLP	6	10	13	14	7	4	0	0	54
Liver rupture	0	1	3	2	4	0	0	0	10
Acute fatty liver	0	0	3	0	0	0	0	0	0
Total	108	145	123	127	83	33	5	1	622

Table 4 shows the percentage of total deaths per level of healthcare due to hypertension. The concern is that although the percentage of deaths may have declined at level one hospitals over the years, the total number of deaths remains high. This may be due to better reporting, but it probably indicates that patients with hypertensive disorders are not being referred to higher levels of healthcare timeously. This finding warrants a review of referral protocols, or barriers to entry to higher levels of healthcare in the provinces. Similarly, the percentage of deaths at each level of healthcare has not changed over the reporting periods.

**Table 4.** Percentage Of Total Deaths Per Level Of Healthcare Caused By Hypertension

*Level*	*2005–2007*			*2002–2004*		
	*Total deaths*	*HT deaths*	*%*	*Total deaths*	*HT deaths*	*%*
Level 1	1429	162	11.3	1103	149	13.5
Level 2	1527	232	15.2	1241	234	18.9
Level 3	850	202	23.8	941	233	24.8

HT: hypertension

## Avoidable factors, missed opportunities and substandard care

Table 5 shows the avoidable factors, missed opportunities and substandard care for hypertension and gives a comparison with the previous reports. There have been improvements in administrative factors but the emergency management of hypertensive disorders remain a concern, particularly at level 1 hospitals. Resuscitation problems have shown a slight decline.

**Table 5.** Avoidable Factors, Missed Opportunities And Substandard Care For Hypertension And Comparison With 2002–2004

*Category*		*Avoidable factors in assessable cases*				
		*2005–2007*		*2002–2004*	
		*n*	*%*	*n*	*%*
Patient orientated		232	42.4	250	47.7
Administrative factors		196	33.4	225	39.3
Health-worker orientated					
Emergency management problems	Level 1	219	65.2	218	65.3
	Level 2	117	34.8	149	51.7
	Level 3	70	32	77	35.6
Resuscitation problems		136	24	148	27.5

Clearly avoidable deaths = 304

Table 6 shows that there were no major differences in the timing of the emergency event. It remains a concern that large numbers of deaths are still occurring in the postpartum period. It ought to be noted that many of the women categorised as having had their emergency event in the postpartum period is arbitrary because they actually had ante- or intrapartum complications, with further complications in the postpartum period. However, 20 women had their first convulsion in the postpartum period, either in hospital or at home following discharge from hospital.

**Table 6.** Timing Of Emergency Event Related To Hypertension

*Category*	*2005–2007*		*2002–2004*	
	*n*	*%*	*n*	*%*
Early pregnancy	14	2.3	13	2.1
Antenatal period	335	53.9	206	48.7
Intrapartum	69	11.1	83	13.2
Postpartum period	209	33.6	228	36.3
Unknown	3	0.5	5	0.8

Patient-orientated problems show slight improvements (Table 7). It is well known that over 90% of women in South Africa do have antenatal care but infrequent attendance and delay in seeking help remain major issues.

**Table 7.** Avoidable Factors, Missed Opportunities And Sub-Standard Care With Respect To Patient-Orientated Problems For Hypertension And A Comparison With 2002–2004

*Major problems*	*% of assessable cases with avoidable factors*			
	*2005–2007*		*2002–2004*	
	*n = 547*	*%*	*n = 524*	*%*
Non-attendance of antenatal care	106	19.4	123	23.5
Infrequent attendance	40	7.3	36	6.9
Delay in seeking help	106	19.4	140	26.7
Other	45	8.2	38	7.3

## Case 1

## Management of severe hypertension in the antenatal period

The patient, a 27-year-old parity 3, had high blood pressure during her antenatal visits. She had had a previous caesarian (C/S) and had three antenatal visits at a tertiary-care centre.

At her last visit, her blood pressure was 170/110 mmHg, she was admitted at 11:00 and methyl dopa was prescribed. She had a seizure at 23:00 on the day of admission. Magnesium sulphate (MgS0_4_) was prescribed and a C/S was performed ‘under platelet cover’. Post-operatively, there was difficulty in controlling her high blood pressure; she had HELLP with jaundice and demised at 12:00 the next day.

## Lessons to learn: immediate management of severe hypertension

Careful history taking and obtaining results of investigations is essential. It is not clear whether information on her past obstetric history was obtained. If she did have a previous C/S for hypertension, laboratory results should have been obtained soon after admission and appropriate action taken timeously. In this case the results were only obtained following her seizure.

It is uncommon, for patients to ‘fit’ under medical management in a tertiary hospital but this case illustrates the fulminant course of pre-eclampsia that some patients develop. Therefore all patients who are admitted with severe hypertension should be treated with urgency.

• Obtain a good past history.
• Lower very high blood pressure reasonably quickly, but in a controlled manner. Dihydralazine, if available, should be used. Alternate antihypertensive agents include labetalol and nifedipine.
• All blood investigations must be reviewed within two to four hours of being sent to a laboratory. This patient had low platelets and HELLP probably on admission and it is likely that her high blood pressure was not controlled.
• Intensive monitoring of blood pressure levels must be performed until they are stabilised.

## Cases 2–4: postpartum convulsions

A 37-year-old delivered a premature baby at a MOU and was transferred to a tertiary hospital because the baby required level 3 care. The patient, on admission to the tertiary hospital, complained of symptoms in keeping with imminent eclampsia and had hypertension. The intern failed to make the correct diagnosis; therefore no observations were carried out. The next day the patient was found in a post-ictal state and subsequently had a cardio-pulmonary arrest.

A 23-year-old, parity 3 had a C/S for foetal distress and imminent eclampsia. On the second post-operative day she developed ‘mild pulmonary oedema’. She responded to initial treatment; 12 hours later, she convulsed and had a cardio-pulmonary arrest.

A parity 1 G 2 had severe pre-eclampsia. She had a normal vaginal delivery but was discharged on day 1 following delivery, with no treatment. She ‘fitted’ at home 24 hours later and had a cardio-pulmonary arrest.

## Lessons to learn

These cases illustrate features of severe pre-eclampsia syndrome that are often not taken into account by inexperienced health personnel. While it is true that in our current understanding of the aetiology of pre-eclampsia that delivery of the foetus and placenta leads to cure, it must be understood that these women are still at risk of complications from the disease process in the immediate post-delivery period. Therefore it is essential to ensure continued frequent observations of the pulse rate, blood pressure, urine output, level of consciousness and potential signs of pulmonary oedema.

Antihypertensive therapy must be continued and should not be stopped abruptly. Magnesium sulphate should be continued for at least 24 hours following delivery. All laboratory tests must be repeated within four to six hours following delivery and results reviewed. Patients should not be discharged until their high blood pressure levels are stabilised for a period of at least 24 hours. Furthermore, these patients should be asked to return to the postnatal clinic within a week and only referred to a clinic when all physical and laboratory tests have returned to normal. Generally speaking, these tests should have returned to normal within seven days of delivery. Advice on contraception, further pregnancies and place of future antenatal care should be provided.

All three cases illustrate the above lessons, namely

• failure to recognise symptoms and signs of imminent eclampsia following delivery and consequently failure of post-delivery observations
• failure to recognise dangers of pulmonary oedema, the need for investigations and frequent observations
• discharging patients from hospital prior to stabilising (lowering) high blood pressure values and continuing antihypertensive therapy.

## Discussion

It is of obvious concern that large numbers of deaths still occur from pre-eclampsia and eclampsia in South Africa. These pregnancy-specific hypertensive conditions are treatable and most are preventable by early detection, adequate treatment and timely delivery. The present report indicates that 304 cases were clearly avoidable.

A recent article^5^ indicates that in poorly resourced countries, the availability of MgS0_4_ and the lack of clinical protocols of management are significant issues associated with maternal deaths due to pre-eclampsia.^5^ In South Africa, these factors should not be issues, as MgS0_4_ is widely available and is on the essential drug list for use at all health facilities. Further, clinical guidelines for the management of hypertension and, in particular, the management of obstetric emergencies have been widely distributed to all hospitals and clinics (Guidelines for Maternity Care in South Africa; Essential steps in the management of common conditions associated with maternal mortality). It does appear though that these guidelines might not be reaching all health professionals. Face-to-face teaching and emergency drills on the labour ward floor, on a regular basis, may improve the situation.

Intracranial haemorrhage remains the commonest final cause of death in hypertensive disorders of pregnancy. Although, the exact mechanisms that are associated with hypertension and intracranial haemorrhage are not clearly understood, it would appear that both systolic and diastolic hypertension play a role. It is generally accepted that diastolic blood pressures of ≥ 110 mmHg are linked with intracranial pathology but recently sustained systolic hypertension has also been found to play a significant role. A systolic blood pressure value above which urgent antihypertensive treatment should be given has been identified as 155–160 mmHg.^6^ In addition, the latest Why Women Die, 2003–2005 publication recommends ‘that women with a systolic blood pressure of ≥ 160 mmHg need antihypertensive treatment. Consideration should also be given to initiating antihypertensive treatment at lower blood pressure values if the overall clinical picture suggests rapid deterioration with anticipation of severe hypertension’.^1^

Clinical experience indicates that in young women with an abrupt onset of severe hypertension, the blood pressure levels are often labile, therefore not only should blood pressure measurements be performed more frequently, but early treatment of hypertension considered. Treatment of very high blood pressure is an essential component of the immediate management of severe hypertension, impending eclampsia and eclampsia, and serious consideration must be given to the use of rapid-acting antihypertensive agents. In South Africa, oral nifedipine, dihydrallazine in some institutions, and intravenous labetalol are available for this purpose.

The commonly used antihypertensive medications for lowering very high blood pressure in pregnancy in South Africa are labetalol, dihydrallazine and nifedipine. These drugs are recommended in the Guidelines for maternity care for clinics and district hospitals.^7^ Parenteral hydralazine and labetalol are not easily available at district hospitals in South Africa. Oral nifedipine is therefore recommended in emergencies to lower very high blood pressure. About 11% of women with hypertensive crises require rapid-acting agents and in order to reduce the hypotensive episodes associated with these medications, it has been recommended that acute high blood pressure be reduced reasonably quickly but in a controlled manner, ideally by slow bolus injections or titration of parenteral drugs. In pregnancy, sodium nitroprusside is only used in an ICU setting or in the operating theatre, therefore the agents most available for use are labetalol, dihydrallazine and nifedipine.^7^,^08^

The latest Saving Mothers report (2005–2007) indicates a number of women developed eclampsia/severe hypertension in the postpartum period. This suggests that antihypertensive medications are stopped once delivery of the baby has occurred. In general, high blood pressures may take up to six weeks to normalise. Therefore, antihypertensive medications must be continued postpartum and the dosage reduced in a stepwise manner.

Cardiac and renal failure were assigned as the final and contributory causes of maternal deaths in 42.9 and 8% of cases, respectively. The number of cases of death due to renal failure has declined but those from cardiac failure have increased since the 2002–2004 report. In the previous report,^3^ it was believed that guidelines for the appropriate fluid balance might be working. However, it is of concern that the number of deaths from cardiac failure (probably pulmonary oedema) has increased, particularly when guidelines on fluid balance are available. In the latest UK report (Why Women Die), there were no deaths from pulmonary causes alone, and this improvement is probably due to the availability and informed use of clinical protocols on good fluid management.^1^

In a recent report on maternal deaths related to hypertension, one of 27 deaths between 2000 and 2004 was due to cardiac failure. The authors point out that postpartum mobilisation of extracellular water and subsequent shift into the intravascular compartment can aggravate hypertension, and that obstetricians should be aware of this.^9^

The categorisation of patients into deaths from renal failure, cardiac failure and respiratory causes of death is to a large extent arbitrary and should not necessarily be viewed as distinct entities. The three entities should be regarded as the same thing, namely fluid overload. Fluid overload should be recognised early and be treated adequately. There is a predominance of deaths from pulmonary oedema in this group of deaths. Therefore, detecting patients at risk by history and examination, early referral and appropriate critical care are essential for this group of patients.

In this audit, of the 207 women who had cerebral complications, the average blood pressure values were systolic 177 (103–244 mmHg) and diastolic 115 mmHg (74–162 mmHg). These are extremely high values and do imply that young women with severe pre-eclampsia and eclampsia require lowering of high blood pressure in a controlled and smooth manner to prevent severe hypotension. This is of particular importance as most of the patients are young and the onset of hypertension is usually abrupt.

Eighty per cent of the 207 women who had convulsions were primigravidae and their mean age was 27 years (range 13–45). Furthermore, 16 had systolic blood pressure values above 160 mmHg with diastolic blood pressures of less than 110 mmHg. High blood pressure control is of extreme importance and due diligence must be given to use of rapid-acting antihypertensive agents and frequent blood pressure measurements in the acute phase of the condition. Attention should also be given to the use of anticonvulsant therapy to arrest seizure activity. It is likely that the blood pressure rises further during seizure activity and that most cerebrovascular accidents occur at this time, particularly in patients with low platelet counts.

Infrequent antenatal attendance continues to be a challenge. In the public hospitals of South Africa, most women ‘book’ for antenatal care but often this is in mid-gestation, and follow-up visits are infrequent. Community education on the benefits of antenatal care was emphasised in the last Saving Mothers report, but it appears that implementation of this recommendation may not have been done. Public health education and involvement of partners and families should, once again, be a priority.

In the previous report, the issue of ‘hidden pregnancies’ was highlighted.^3^ In this report a large proportion of young women died from eclampsia.^2^ In particular, three teenagers aged 13, 14 and 15 years stand out. All had hidden pregnancies, therefore there was considerable delay in seeking help. Education, provision of contraception and family support are key factors in preventing teenage pregnancies

Maternity care is free of financial cost in South Africa and access to antenatal clinics widely available, therefore other issues, social and cultural factors may be involved. Improvement in education in general, the dissemination of health information on radio, television and the print media may be ways of overcoming this challenge. More specifically, education of antenatal attendees at time of booking and at every visit should be emphasised. Women with chronic hypertension or those pre-eclamptics being treated as outpatients should be informed about the likely complications and steps to take if these symptoms occur.

The previous report also highlighted the problems related to emergency management and recommendations were made regarding under-, post-graduate and continuing professional education, short of instituting that all doctors should undergo a course on emergency resuscitation prior to registering as general practitioners. Hands-on courses on mannequins done at frequent intervals are also recommended. The National Committee on Confidential Enquiries into Maternal Deaths is promoting a training programme on obstetric emergencies called Essential Steps in the Management of Obstetric Emergencies (ESMOE). This involves trainers visiting all health districts and carrying out face-to-face training for doctors on mannequins and other visual aids on the emergency management of common conditions causing maternal deaths.

## Conclusion

Deaths from hypertensive disorders of pregnancy remain the commonest direct cause of maternal death and most of the deaths had avoidable factors and substandard care. There is a continuing need for better education of women, families, communities and professionals concerning the danger signs of the complications of hypertension. The need for early and regular attendance of antenatal care should also be emphasised. In addition, health professionals need to be educated about women at risk and be aware of factors associated with complications associated with pregnancy hypertension (Table 8).

**Table 8.** Be Aware / Be Alert

Preventing cerebrovascular accidents:
• Treat sustained systolic hypertension (≥ 160 mmHg)
• Treat fluctuating (very high levels) blood pressure (≥ 160 mmHg). Lower high blood pressure smoothly with IV antihypertensive agents
• Need for continuing close blood pressure monitoring; continuing antihypertensive drugs in the immediate postpartum period
• The use of MgS0_4_ in severe fulminant hypertension and its continued use for up to 24 hours following delivery
Preventing pulmonary oedema:
• Fluid overload: watch out for early signs
Continuing education:
• Face-to-face training on obstetric emergencies and the use of obstetric drills
